# Post-tubercular Left Lung Destruction With Compensatory Hyperinflation of the Right Lung: A Complicated Case

**DOI:** 10.7759/cureus.64904

**Published:** 2024-07-19

**Authors:** Adish Kurdukar, Pankaj Wagh, Sanvi Arora, Kanishk Shukla, Souvik Sarkar

**Affiliations:** 1 Respiratory Medicine, Jawaharlal Nehru Medical College, Datta Meghe Institute of Higher Education and Research, Wardha, IND

**Keywords:** lung volume reduction surgery, bronchodilators, post-tb, tuberculosis, compensatory hyperinflation

## Abstract

Hyperinflation is the rise in functional residual capacity, i.e., the volume of air left in the lung after normal expiration. One lung is wholly damaged and nonfunctional, while the other lung increases its surface area to compensate for the loss of the respiratory system. Pulmonary tuberculosis (TB) is a respiratory disease caused by *Mycobacterium tuberculosis*, primarily targeting the lungs. However, if left untreated, it could lead to life-threatening conditions, such as systemic manifestations, and increase the mortality rate. When TB causes severe damage to one lung, the other lung may compensate by hyperinflating excessively to keep the body's oxygenation levels healthy. It was seen in the case of a 60-year-old male who presented to the Outpatient Department (OPD) with complaints of hearing loss, blood-tinged sputum, and cough. In investigations, compensatory hyperinflation was seen. TB and hyperinflation of the lung are not associated together, and hyperinflation is not a clinical sign of TB. This distinction is what distinguishes this particular case.

## Introduction

Tuberculosis (TB) is a public health problem of great concern, even after numerous attempts made by the World Health Organization and its partners [1]. TB is caused by *Mycobacterium tuberculosis*, which is transmitted airborne when a TB patient coughs or sneezes. TB is said to affect multiple systems of the body and has many manifestations, such as pulmonary, hepatobiliary, genitourinary, spinal, and intestinal. Post-tubercular left-sided lung destruction with a compensatory hyperinflated right lung is a compensatory mechanism where the functional lung expands to compensate for the loss of respiratory function in a damaged lung. In post-TB complications, the body tries to compensate for damage caused by hyperinflation in the unaffected areas of the lungs, which may lead to over-distention of the remaining lung [2]. Numerous diseases, including bronchiectasis, emphysema, cystic fibrosis, and postoperative management of single lung transplant surgery, can cause TB [3]. A proper analysis is needed to diagnose post-TB hyperinflation, which is done by chest X-rays to assess the structural changes and pulmonary function tests (PFTs) to measure lung volumes and capacities [4]. TB and hyperinflation of the lung are not linked together, and hyperinflation is not a clinical sign of TB. This distinction is what distinguishes this particular case.

## Case presentation

**Figure 1:**
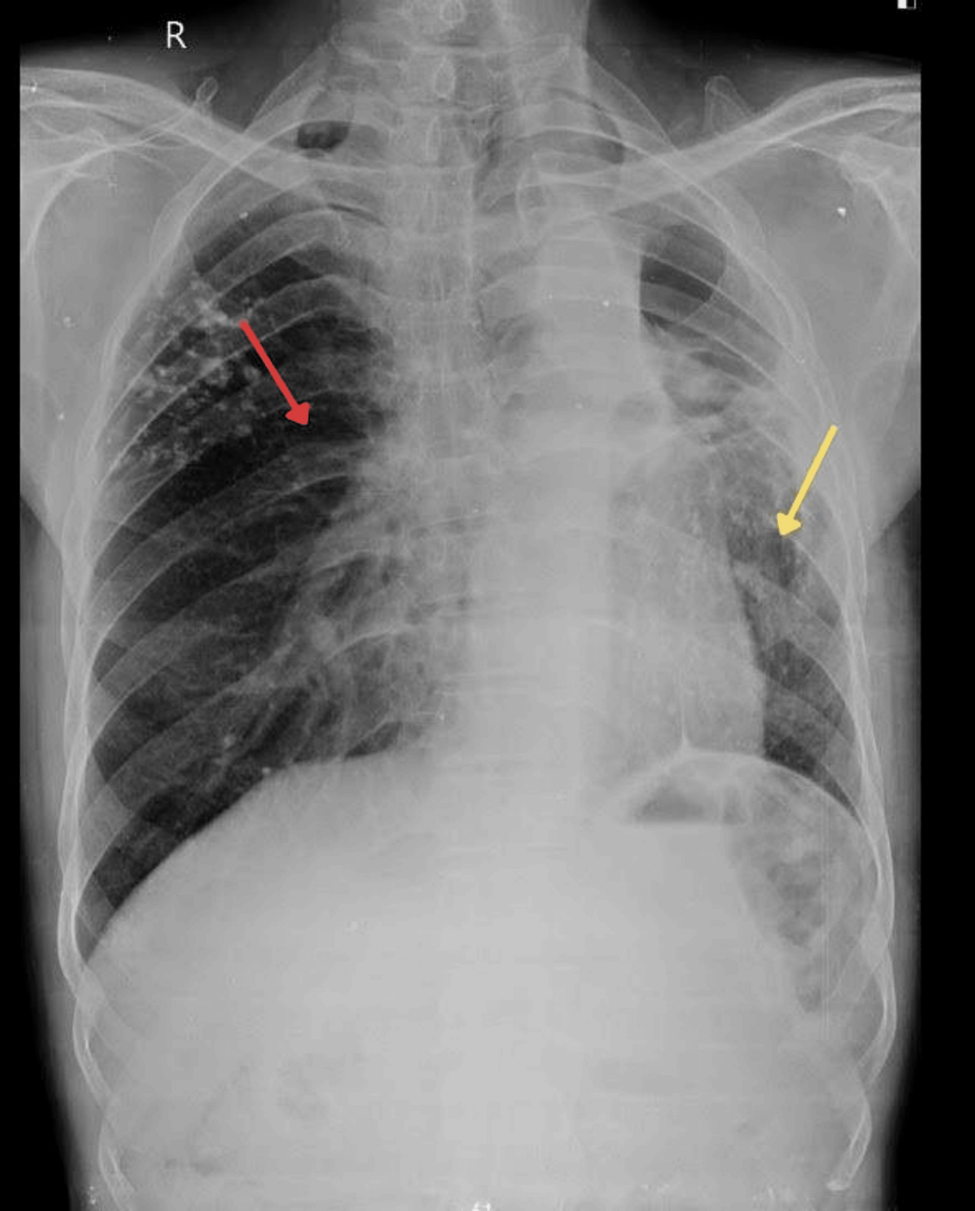
The X-ray AP view shows a destroyed left lung (yellow arrow) and a hyperinflated right lung (red arrow). AP: anterior posterior

A 60-year-old male presented to the Outpatient Department (OPD) with complaints of blood-tinged sputum for six years, which was associated with hearing loss and coughing. There was a relevant history of TB for which the patient took Protein Kinase B (AKT) therapy for three years. The patient was advised for a chest X-ray, which showed evidence of nodular infiltration of both lungs with multiple calcific foci (Figure 1).

**Figure 2:**
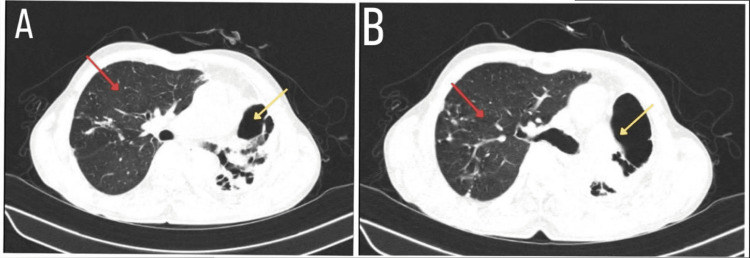
The HRCT shows a destroyed left lung (yellow arrow) and a compensatory hyperinflated right lung (red arrow) in both A and B. HRCT: high-resolution computed tomography

An elevation of the right hemidiaphragm was observed, along with a cavitary lesion in the right upper lobe. High-resonance contrast tomography (HRCT) of the thorax had the findings of extensive areas of cystic bronchiectasis noted in the left upper lobe with significant volume loss of the left upper lobe, multiple calcific foci noted in bilateral lung fields predominantly in the correct upper and left lower lobe, and compensatory hyperinflation of proper lung fields (Figure 2).

In response to the history of blood-tinged sputum, previous history of TB, and chest X-ray findings, all were suggestive of post-TB complications. He complained of right-sided chest pain, cough with mucoid expectorations, occasional bloody sputum, and breathlessness on exertion. There was no history of fever, cold, orthopnea, or paroxysmal nocturnal dyspnea. The patient’s appetite was normal, with a weight loss of 3 kg. A history of tobacco consumption of more than 40 years is present, with a history of pulmonary TB in 2010. Treatment was continued until 2013, but no relevant records are available.

On general examination, the patient had tachycardia at 110/minute, tachypneic with 20/minute thoracoabdominal, blood pressure of 100/60 mmHg, and pulse oximetry showed 93% saturation. The nervous and gastrointestinal systems were standard during the clinical examination, but the respiratory system revealed findings. Left bronchial breath sounds were heard, and crepitus was on the right side. A provisional diagnosis of post-TB hyperinflated lung sequelae was given, and reactivation of pulmonary Koch was suspected. The patient was further advised to undergo an HRCT of the sputum examination for acid-fast bacilli, 2D echocardiography, impulse oscillometry (IOS), fibreoptic bronchoscopy (FoB), and triple H antigen test. The pulmonary function test indicated an FEV1/FVC ratio of more than 70% and an FEV1 ratio of less than 80%, indicative of a mixed pattern exhibiting an obstructive and restrictive nature of lung disease.

## Discussion

TB leads to several chronic diseases, such as chronic obstructive pulmonary disease (COPD), bronchiectasis, pulmonary fibrosis, cor pulmonale, and right-side failure. Hyperinflation is a complication of TB in which there is a rise in the functional residual capacity, i.e., the volume of air left in the lung after normal expiration [5]. One lung is wholly damaged and nonfunctional; the other lung increases its surface area to compensate for the loss of the respiratory system. The lung parenchyma is destroyed in the end stage of TB [6]. This compensatory mechanism enables the unaffected lung to exchange more oxygen and carbon dioxide to mitigate some of the symptoms of reduced lung function.

Several mechanisms cause post-TB hyperinflation, such as scarring and fibrosis. Granulomatous inflammation brought on by TB leads to fibrosis formation; during the healing process, scarring occurs, which causes complete or partial obstruction by severely narrowing the airways, especially the smaller bronchioles. Consequently, air becomes trapped in the alveoli during exhalation, exacerbating hyperinflation [7]. In some cases, TB causes emphysema, which involves destroying the alveolar walls, reducing the gas exchange, and contributing to hyperinflation. When compensatory hyperinflation of the normal lung segment occurs, a mediastinal shift pulls up the diaphragm and retracts the helium.

The clinical symptoms of post-TB hyperinflation vary from patient to patient. Dyspnea is the most common symptom, which can limit physical activity and exercise tolerance. Patients also experience mild-to-severe chest tightness and discomfort, which can be exacerbated by performing physical activity. These symptoms may occur due to obstruction and loss of elasticity in the lung, leading the chest to expand beyond its normal capacity and causing a feeling of tightness [8]. It can also be caused by the flattening of the diaphragm, which impairs its ability to expand and contract properly [9].

Our case study shows compensatory hyperinflation of the right lung in post-tubercular patients, which is quite complex, unusual, and rare because hyperinflation of the lung is not a clinical indication of the disease [10]. From 1999 to 2014, 13 patients from Spain had the same complications and symptoms, such as hemoptysis, as this patient [11]. The only difference is that they had TB since childhood and middle age, whereas this patient had TB three years ago, making this case different from previously published cases [12].

Therapeutic interventions include improving the right lung and lung function and managing symptoms. Bronchodilators are given, which relax smooth muscle tone in the airway, leading to enhanced ventilatory mechanisms. Among the several bronchodilators frequently prescribed for COPD are methylxanthines, beta-agonists, and anticholinergics. Beta-agonists stimulate beta-adrenergic receptors, leading to the relaxation of smooth muscles and causing bronchodilation. Methylxanthines, like theophylline, function by blocking phosphodiesterase, which raises cyclic adenosine monophosphate (cAMP) in the smooth muscle cells of the airways, which causes bronchodilation and smooth muscle relaxation. Anticholinergics block the action of acetylcholine, preventing the contraction of airway smooth muscle [13]. Pulmonary rehabilitation is one of the interventions in which there is a decrease in carbon dioxide production and increasing oxygen uptake. Patients with severe hyperinflation of the lung, like this patient, will have limited respiratory changes [14]. Surgical interventions such as lung volume reduction surgery (LVRS) or lung transplantation are recommended. LVRS involves removing a damaged portion of the lung to improve breathing mechanisms. In a condition where hyperinflation occurs with asthma, the use of anti-inflammatory drugs can also be seen [15].

## Conclusions

TB is a significant health problem that results in several chronic diseases, such as chronic obstructive pulmonary disease, bronchiectasis, pulmonary fibrosis, cor pulmonale, and right-side failure. Hyperinflation is one complication of TB where there is a rise in the functional residual capacity. This case involved a 60-year-old male patient coming to the hospital with complaints of blood-tinged sputum, which were all suggestive of TB complications. The patient was also found to have compensatory lung hyperinflation on HRCT, along with other notable findings. However, TB and hyperinflation of the lung are not linked, and hyperinflation is not a clinical sign of TB, which is what distinguishes this particular case. A provisional diagnosis of post-TB sequelae was given, and the reactivation of pulmonary Koch was considered.
